# Genome Sequence of *Halobacterium* sp. Strain BOL4-2, Isolated and Cultured from Salar de Uyuni, Bolivia

**DOI:** 10.1128/MRA.01045-21

**Published:** 2021-12-02

**Authors:** Priya DasSarma, Brian P. Anton, Hedvig A. L. von Ehrenheim, Fabiana L. Martinez, Daniel Guzmán, Richard J. Roberts, Shiladitya DasSarma

**Affiliations:** a Institute of Marine and Environmental Technology, Department of Microbiology and Immunology, University of Maryland, Baltimore, Maryland, USA; b New England Biolabs, Ipswich, Massachusetts, USA; c Centro de Biotecnología, Faculty of Sciences and Technology, Universidad Mayor de San Simón, Cochabamba, Bolivia; Portland State University

## Abstract

*Halobacterium* sp. strain BOL4-2 was isolated from an Andean salt flat, Salar de Uyuni, in Bolivia. Single-molecule real-time (SMRT) sequencing revealed a 2.4-Mbp genome with a 2.0-Mbp chromosome and four plasmids (2 to 299 kb). Its isolation from an environment experiencing multiple extremes makes the strain interesting for astrobiology.

## ANNOUNCEMENT

An extreme halophile, strain BOL4-2, was isolated from Salar de Uyuni, Department of Potosí, Bolivia, the world's largest salt flat and an environment remarkable for its high elevation, cold temperatures, and UV radiation exposure (1–4). Such environments are of significant interest to the astrobiology community due to their multiple extremes (4–11).

For isolation, salt crust was sampled at the surface of Salar de Uyuni (20°33′28.58″S, 67°12′29.56″W), 3,647 m above sea level, in March 2015. Typical conditions there include pH values of 7.3 to 7.6, ≥28% (wt/vol) NaCl concentration, and temperatures ranging from −15 to 22°C. Salt samples were dissolved in complete medium plus trace metals (CM^+^), and cells were grown at 37°C with shaking at 220 rpm (product number 4230; Innova, New Brunswick, NJ, USA) as described previously (12, 13). The enrichment culture was plated on CM^+^ agar plates and purified by three rounds of streaking.

Nucleic acids were extracted using standard methods (14–16). Briefly, after cell lysis by osmotic shock, the lysate was extracted three times with phenol saturated with Tris-HCl (pH 8), followed by dialysis and treatment with RNase. Single-molecule real-time (SMRT) sequencing was performed using the Sequel platform (Pacific Biosciences [PacBio], Menlo Park, CA). Genomic DNA (2 μg) was randomly sheared with a Megaruptor (Diagenode, Denville, NJ) using the 40-kb setting, and a SMRTbell sequencing library was prepared from the sheared DNA using a modified version of the manufacturer’s protocol (17). No size selection was performed. The library was sequenced on a single SMRT cell (Sequel binding kit v3.0 and Sequel sequencing plate v3.0) with 10-h collection and 2-h preextension times. Sequencing reads were filtered (quality scores of ≥0.7) and assembled (2,485,291 subreads [mean length, 4,516 bp]) separately with HGAP4 (using FALCON override ovlp_DBsplit_option = -s50, yielding 7 contigs) and Microbial Assembly analysis (using default parameters, yielding 16 contigs). Both sets included 5 contigs with high levels of coverage (∼4,000×), which formed the final assembly. Three contigs were circularized automatically with Microbial Assembly analysis, a fourth (pBOL4-2_299.7) was circularized from an HGAP4 contig and checked by manual analysis of read structure mapped back to the circularization point, and the fifth (pBOL4-2_2.1) manifested as a linear concatemer in both assemblies, with the final sequence inferred as the monomer. Methylation patterns were determined using Base Modification analysis using default parameters. All PacBio programs were run under the SMRT Link v6.0.0.47841 environment.

Genome annotation was performed in house using GeneMark.hmm v2 (18) and EMBOSS (19). The genome was also processed through the NCBI Prokaryotic Genome Annotation Pipeline (PGAP) (build 3190) (20).

The 2,428,492-bp genome consists of a large circular chromosome (1,989,773 bp [GC content, 68.1%]) and four plasmids, i.e., pBOL4-2_299.7 (299,663 bp [GC content, 59.1%]), pBOL4-2_105.9 (105,947 bp [GC content, 60%]), pBOL4-2_49.8 (49,847 bp [GC content, 61.5%]), and pBOL4-2_2.1 (2,144 bp [GC content, 61.5%]). A single rRNA operon, including 16S rRNA and 47 tRNA genes, are present. The genome sequence was submitted to GenBank, and the taxonomy was determined by NCBI Taxonomy (21).

The *Halobacterium* sp. strain BOL4-2 genome contained 2,555 encoded proteins, with a calculated mean pI value of 4.91, a highly acidic proteome characteristic of haloarchaea (22–24). The genome encodes all 799 conserved core haloarchaeal groups (cHOGs) (25), 10 Orc/Cdc6 proteins, a TATA-binding protein, and 7 transcription factor B (TFB) proteins (26–29). Also encoded are the retinal proteins bacteriorhodopsin, halorhodopsin, and sensory rhodopsin 2 (30) and buoyant gas vesicle nanoparticles (31, 32). The methylated DNA motifs (with methylated base underlined) recorded in the REBASE database include CTAG (m4C), GTCACG (m4C), CGAYNNNNNNGTRC/GYACNNNNNNRTCG (m6A), and AGCANNNNNNCTG/CAGNNNNNNTGCT (m6A) (33).

### Data availability.

The *Halobacterium* BOL4-2 genome sequence has been deposited in GenBank with the accession numbers CP070332.1, CP070334.1, CP070335.1, CP070336.1, and CP070337.1. Raw data are available in the NCBI Sequence Read Archive (SRA) with the accession number SRX10292502.
